# Early-Career Systems Microbiology Scientists

**DOI:** 10.1128/mSystems.00002-18

**Published:** 2018-03-06

**Authors:** Jack A. Gilbert

**Affiliations:** a Biosciences Division, Argonne National Laboratory, Argonne, Illinois, USA; b The Microbiome Center, Department of Surgery, University of Chicago, Chicago, Illinois, USA

## EDITORIAL

Early-career scientists, especially those just stepping into their first independent roles, represent the tenacity of our creed to continue exploring the microbial world through structured questioning, testing, and observation. While sometimes confused with the epithet “young scientist,” which may represent people with either considerable or limited experience, those in their early career are just starting to craft their professional reputation. This is a most exciting time; all of us can remember that first time we sat down at a desk or bench and realized that for all intents and purposes we were now responsible for our own fate. Let loose from the comparative shackles of graduate school and postdoctoral martyrdom, this period in our growth as professional scientists often presents so many opportunities that it can be bewildering.

Investigators in this early phase of development are also at one of the most awe-inspiring periods in their careers. The infusion of fresh ideas that come in part from the willingness to challenge the *status quo* means that this stage can often deliver some of the most exciting new ideas in the field. The bounty of optimism, creativity, and energy can lead to phenomenal productivity and the development of revolutionary ideas. Too often the established dogma of the “late-career” scientists can be hard to shift, which can result in early-career scientists trying to play in the same sandbox. However, what defines this career phase is that you can break out, harness your enthusiasm to deconstruct and rebuild, or completely change direction. Those who do this, more often than not, are recognized as contributing to paradigm shifts in the domain. Assistant professor-level researchers are often overlooked, especially at conferences, where we end up hearing from the “famous” scientists again and again. However, those in this career stage are an immense resource and have a huge amount to offer. We should value them more than we currently do and provide them with a voice that may help energize our scientific family to embrace a diversity of thought.

When we write mentoring plans for the postdoctoral stage of career development, we often say that the postdoctoral stage is instrumental as students transition to more independent roles, crafting new skills and preparing to move forward for tenure-track or other professional jobs. This phraseology could easily be applied to every stage of career development. If the postdoctoral stage is supposed to help train you to be independent, to acquire those skills that will allow you to manage people, balance budgets, make decisions, and push back the frontiers of science, then the early-career scientist is supposed to embody these ideals. As early-career scientists, we are quickly taught that financial planning and security should be our main aim, but we also have to publish or produce work that is recognized in our respective fields, both within our institution and without. How do we achieve this complex balance? This is hard enough when you reach a career level that provides support such as personal assistants, grant writers, travel and financial administrators, and all those wonderful people who make day-to-day work life possible. But without those trappings, the burden of new responsibility can be daunting. So, it is all the more commendable when early-career scientists are able to build an incredible reputation within the first few years of their new position.

This special issue should be considered a guidebook to some of the best and brightest early-career minds currently at work in microbial systems biology. We wanted to provide a forum to highlight what some of the incredible innovators in our community are doing and where those who embody the future of our field see their science progressing over the coming years. The special issue was open to anyone who wanted to submit an article, but many people were encouraged to submit by leaders in the field, a testament to their burgeoning reputation. We are very excited to provide this special issue, which we will revisit periodically, as a veritable who’s who of systems microbiology. We hope that you enjoy the ideas, concepts, and visions presented here.

